# ANNOUNCEMENTS & RESOURCES

**Published:** 2018-11-09

## Emergency eye care

In the past, eye emergencies were often dealt with by the most junior members of the team. Now, there is a growing awareness that these emergencies are dealt with better when overseen by a more senior consultant. The British Emergency Eye Care Society was founded in 2013 to bring together practitioners who provide acute ophthalmology services in the UK to improve practice and promote excellence in patient care. To find out more, visit **www.beecs.co.uk**

**Figure:**
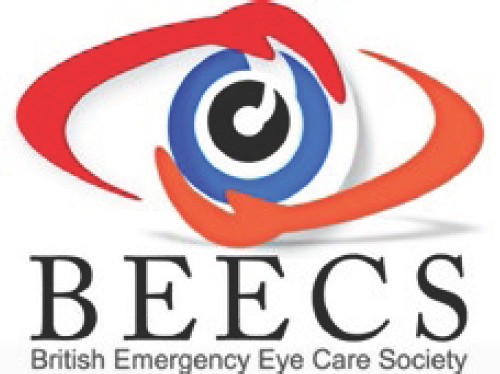


## IAPB Council of Members meeting 2018

A total of 350 members of over 100 global and local eye health organisations recently met in India at the International Agency for the Prevention of Blindness (IAPB) Council of Members meeting in Hyderabad, India. On the agenda was Universal Health Coverage and eye health, technology, and partnerships in eye care. A round-up with videos and photos from the different meetings are available online at **http://bit.ly/CoM18RP**

**Figure:**
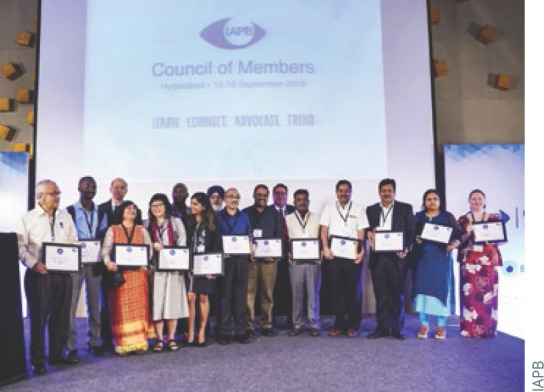


## Courses

### MSc Public Health for Eye Care, London School of Hygiene & Tropical Medicine

Fully funded scholarships are available for Commonwealth country nationals. The course aims to provide eye health professionals with the public health knowledge and skills required to reduce blindness and visual disability. For more information visit **www.lshtm.ac.uk/study/masters/mscphec.html** or email **romulo.fabunan@lsthm.ac.uk**

#### Free online courses

**The ICEH Open Education for eye care programme** offers a series of online courses in key topics in public health eye care. All the courses are free to access and include: Global Blindness, Eliminating Trachoma, Ophthalmic Epidemiology: Basic Principles and Application to Eye Disease.

More free courses coming! Certification also available.

For more information visit **http://iceh.lshtm.ac.uk/oer/**

## Subscriptions

Contact Anita Shah **admin@cehjournal.org**

### Subscribe to our mailing list

**web@cehjournal.org** or visit **www.cehjournal.org/subscribe**

### Visit us online

**www.cehjournal.org www.facebook.com/CEHJournal https://twitter.com/CEHJournal**

